# Development and internal validation of a model to predict type 2 diabetic complications after gestational diabetes

**DOI:** 10.1038/s41598-022-14215-9

**Published:** 2022-06-20

**Authors:** Ugochinyere Vivian Ukah, Robert W. Platt, Nathalie Auger, Kaberi Dasgupta, Natalie Dayan

**Affiliations:** 1 Department of Epidemiology, Biostatistics, and Occupational Health, McGill University, Montreal, QC Canada; 2 Institut National de Santé Publique du Québec, Montreal, QC Canada; 3 Lady Davis Institute for Medical Research, Jewish General Hospital, Montreal, QC Canada; 4 Department of Pediatrics, McGill University, Montreal, QC Canada; 5 University of Montreal Hospital Research Centre, Montreal, QC Canada; 6 Department of Social and Preventive Medicine, School of Public Health, University of Montreal, Montreal, QC Canada; 7 Department of Medicine, McGill University Health Centre, Montreal, QC Canada; 8 Department of Obstetrics and Gynecology, McGill University Health Centre, Montreal, QC Canada

**Keywords:** Diabetes complications, Gestational diabetes, Type 2 diabetes, Population screening

## Abstract

Gestational diabetes mellitus (GDM) increases the risk of early-onset type 2 diabetes, which further exacerbates the risk of developing diabetic complications such as kidney, circulatory, and neurological complications. Yet, existing models have solely focused on the prediction of type 2 diabetes, and not of its complications, which are arguably the most clinically relevant outcomes. Our aim was to develop a prediction model for type 2 diabetic complications in patients with GDM. Using provincial administrative data from Quebec, Canada, we developed a model to predict type 2 diabetic complications within 10 years among 90,143 women with GDM. The model was internally validated and assessed for discrimination, calibration, and risk stratification accuracy. The incidence of diabetic complications was 3.8 (95% confidence interval (CI) 3.4–4.3) per 10,000 person-years. The final prediction model included maternal age, socioeconomic deprivation, substance use disorder, gestational age at delivery, severe maternal morbidity, previous pregnancy complications, and hypertensive disorders of pregnancy. The model had good discrimination [area under the curve (AUROC) 0.72 (95% CI 0.69–0.74)] and calibration (slope ≥ 0.9) to predict diabetic complications. In the highest category of the risk stratification table, the positive likelihood ratio was 8.68 (95% CI 4.14–18.23), thereby showing a moderate ability to identify women at highest risk of developing type 2 diabetic complications. Our model predicts the risk of type 2 diabetic complications with moderate accuracy and, once externally validated, may prove to be a useful tool in the management of women after GDM.

## Introduction

Gestational diabetes mellitus (GDM) is a common metabolic disorder of pregnancy, affecting up to 5–14% of pregnant women^1–3^. GDM is associated with approximately 20% greater risk of type 2 diabetes in the decade following delivery^4,5^. Early onset of type 2 diabetes is a risk factor for diabetic complications such as renal disease, circulatory disease, and diabetic acidosis, which pose huge public health concern^6,7^. Guidelines from national diabetes associations in Canada and the US^8,9^ recommend screening for type 2 diabetes 6 weeks to 6 months after a pregnancy complicated by GDM. However, this recommendation has been challenging to implement as less than 50% of women with GDM are screened after pregnancy^10,11^. As the majority of women who have GDM do not develop type 2 diabetes in the short term, targeting high-risk individuals through a risk score may increase screening and interventions to prevent future diabetic complications.

A few models exist to predict the development of type 2 diabetes in women with GDM^12,13^. These models, however, focus solely on the development of type 2 diabetes, and do not include diabetic complications—arguably the more clinically relevant downstream outcomes accounting for the highest economic burden due to diabetes mellitus^6^. Furthermore, the small sample sizes of these studies (less than 500 individuals) limit the generalizability of their findings and model application. There are currently no existing models to predict the risk of type 2 diabetic complications after GDM. There is, therefore, a need for a simple model to help with identifying women with GDM who are at the most risk of developing type 2 diabetic complications, and improve targeted surveillance. Our objective was to develop a model using large administrative data to predict the risk of developing type 2 diabetic complications occurring within 10 years of delivery, among women with a history of GDM. We hypothesized that such easily measured factors can reasonably predict these adverse outcomes in women with GDM and be used to guide health policy planning on a population level.

## Materials and methods

### Ethics

The data used for this study were de-identified and ethics review and participant consent were waived by the institutional review board of the University of Montreal Hospital Centre. All methods were carried out in accordance with the Tri-Council Policy Statement: Ethical Conduct for Research Involving Humans.

### Study population

We conducted a retrospective cohort study of women who had hospital-based deliveries in Quebec, Canada from April 1989 until March 2016 (cohort entry); women were then followed until 2018 to identify outcomes^14^. The cohort was constructed from the Maintenance and Use of Data for the Study of Hospital Clientele registry, which comprises > 99% of deliveries in Quebec.

Individuals aged 18 to 45 years who had GDM in at least one pregnancy were included, with the cohort entry point (t_0_) at the first GDM-affected pregnancy. GDM was defined as abnormal glucose tolerance of the mother, first identified during pregnancy, and identified using diagnostic codes from the 9th and 10th revisions of the International Classification of Diseases (ICD) (Table S1). These codes have been previously validated and adequately capture GDM diagnoses with specificity of > 90% and positive predictive values of > 80%^15,16^. There are some variations in approaches for identifying GDM in different centres, that is, one-step vs two-step approaches; however, both approaches are endorsed by Diabetes Canada^17^.

**Figure 1:**
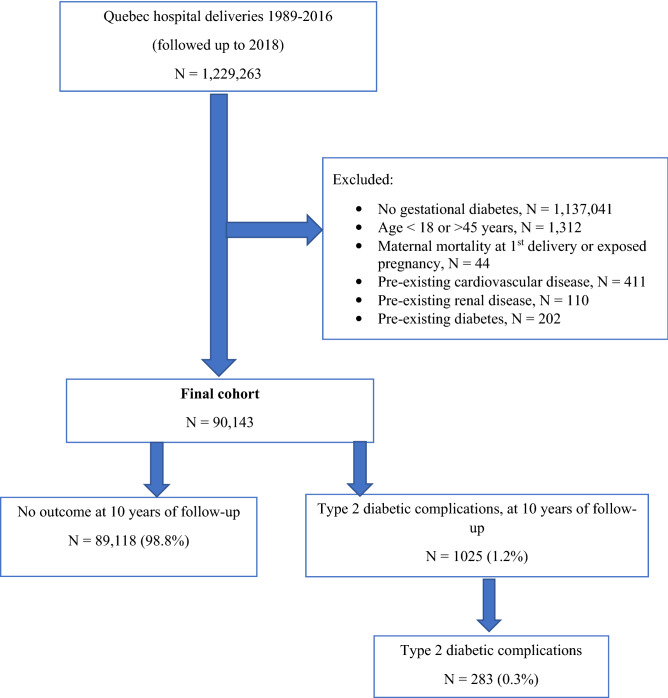
Development of study cohort.

Women who died in their first affected pregnancy and women with pre-existing diabetes, or its complications were excluded (Fig. 1).

### Outcome

The primary outcome was hospitalization for type 2 diabetic complications within 10 years after delivery of the first pregnancy affected by GDM. Type 2 diabetic complications was defined as a diagnosis of type 2 diabetes with the development of one or more of the following complications: diabetic coma, acidosis, kidney, ophthalmic, neurological, circulatory, or other complications resulting from diabetes and identified using ICD-9 and 10 codes, previously validated in studies with specificity of 99% and positive predictive values of > 80% (Table S1).

The secondary outcome was type 2 diabetic complications occurring anytime (up to 29 years) after delivery of the first pregnancy affected by GDM.

Women were followed from cohort entry until any of the outcome occurrence, death, or the end of the study period (March 31, 2018).

### Statistical analyses

We developed Cox proportional hazards regression models to predict type 2 diabetic complications, according to the previously outlined steps^18,19^, and report the process using the Transparent Reporting of a multivariable prediction model for Individual Prognosis Or Diagnosis (TRIPOD) guidelines (Table S2)^20^.

### Candidate predictors, variable selection and coding

We considered demographic, reproductive, and clinical factors known to be associated with an increased risk of type 2 diabetes as potential predictor variables^5,21^. These factors included maternal age, substance use, morbid obesity, socioeconomic deprivation (measured using a composite score of neighbourhood income, education, and employment)^22^, pregnancy factors such as parity, and multifetal pregnancy, and pregnancy complications such as hypertensive disorders of pregnancy (HDP), severe maternal morbidity (SMM)^23^, stillbirth, preterm delivery, low birth weight, and admission into neonatal intensive care unit (NICU) or adult intensive care unit (ICU). Candidate predictors were measured at the time of the index delivery (cohort entry).

Clinical variables that had low incidence were combined with other similar variables (e.g., previous obstetric complications such as SMM, stillbirth, preterm delivery, low birthweight, NICU admission, or neonatal death were combined). Previous history of obstetric complications was further combined with parity as follows: previous obstetric complication (among multiparous women), no previous obstetric complication (among multiparous women) and no previous obstetric complication (among primiparous women). When collinearity (r > 0.5) existed between variables, the most clinically relevant variable was selected.

Continuous candidate predictor variables (e.g., maternal age) were modelled using restricted cubic splines with three knot locations^19^. We assessed interaction terms and retained predictors that were statistically significant (alpha = 0.10)^18^. The final model variables were selected using Least Absolute Selection and Shrinkage Operator (LASSO) regression^18^.

### Model performance and internal validation

Predictive performance of the model was assessed based on discriminatory, calibration, and risk stratification accuracy^18^. Discrimination was measured by the *c*-statistic, which is equivalent to the area under the receiver operating characteristic curve (AUROC)^19^. An AUROC of ≥ 0.7 was interpreted as good discrimination and 0.6 to < 0.7 modest, while 0.5 to 0.6 was considered poor and < 0.5 as not having any discriminative ability. Calibration performance was examined by plotting the mean observed events versus the mean predicted risks by decile. Calibration slopes were interpreted as good (slope > 0.7), poor (0.5 < slope ≤ 0.7) or non-informative (slope ≤ 0.5)^24^.

Using a risk classification table, we examined the ability of the model to stratify the population into low- and high-risk categories. We divided the population into four risk groups, with the highest calculated risk group corresponding to the overall incidence rate of the outcome in the study population^25^. Likelihood ratios (LR) were computed to assess the classification accuracy within each group^26^. For clinical use, positive LRs (LR+) of > 5 or > 10 were interpreted as moderate or good “rule-in” tests, respectively, whereas negative LRs (LR−) of < 0.2 and < 0.1 were considered as moderate or good rule-out tests, respectively^24^.

The model was assessed for internal validity using the bootstrap method with 200 iterations and the over-optimism (i.e., degree to which a model is overfit) was reported^18^.

### Secondary analyses

Using the same final selected variables, we also developed a prediction model for type 2 diabetic complication up to 29 years after delivery and assessed the model discriminatory performance.

### Sample size

We estimated our sample size based on the rule of thumb of 10–20 events per degree of freedom^19^, to avoid model overfitting. With a total of 1025 events during follow-up, we had sufficient sample size to consider up to 50 degrees of freedom for candidate predictors.

Analyses were conducted using R version 3.5.1 (The R Project for Statistical Computing).

## Result

### Cohort description

Among 1,229,263 women who delivered between 1989 and 2016 in Quebec, our final cohort included 90,143 (7.3%) individuals who met study inclusion criteria (Fig. 1). Among these individuals, the number of people admitted to hospital within 10 years of delivery with any diagnosis of type 2 diabetes, including both complicated and uncomplicated diabetes, was 1858 (2.06%) corresponding to an incidence rate of 25.0 (23.9–26.2) per 10,000 person-years. Within 10 years of delivery, there were 283 (0.3%) women who were hospitalized with type 2 diabetic complications [incidence rate 3.8 per 10,000 person-years (95% CI 3.4–4.3)], (Table 1). The median follow-up time was 6.2 years. The incidence of type 2 diabetic complications was higher for women who were younger than 25 years, obese, had substance use disorders, or were socioeconomically deprived at their first GDM-affected pregnancy, compared with those who did not have these characteristics (Table 1).

**Table 1:** Ten-year incidence of type 2 diabetic complications in women with gestational diabetes mellitus, according to characteristics at cohort entry.

	Total number of women	Incidence per 10,000 person-years (95% confidence interval)
**Age, years**		
< 25	10,912	7.1 (5.5–9.0)
25–29	25,703	3.7 (3.0–4.6)
30–34	30,666	3.1 (2.5–3.9)
≥ 35	22,862	3.1 (2.3–4.0)
**Primiparity**		
Yes	54,342	3.9 (3.3–4.5)
No	35,801	3.7 (3.0–4.5)
**Morbid obesity**		
Yes	4070	13.6 (9.4–19.1)
No	86,073	3.5 (3.1–3.9)
**Substance use**^**a**^		
Yes	1684	7.2 (3.3–13.6)
No	88,459	3.8 (3.3–4.2)
**Socioeconomic deprivation**		
Deprived	20,432	7.0 (5.8–8.4)
Not deprived	65,308	2.8 (2.4–3.3)
**Time period**		
1989–1995	17,250	2.0 (1.4–2.7)
1996–2002	17,292	3.5 (2.7–4.5)
2003–2009	23,036	5. 5 (4.5–6.5)
2010–2016	32,565	4.1 (3.1–5.2)
**HDP type**		
No HDP	80,858	3.3 (2.9- 3.8)
Pre-existing or unspecified hypertension	3035	6.2 (3.6–9.9)
Gestational hypertension	2864	7.5 (4.3–12.2)
Pre-eclampsia/HELLP syndrome	3058	9.6 (6.2–14.3)
Superimposed pre-eclampsia	328	12.4 (2.6–36.3)
Total	90,143	3.8 (3.4–4.3)

^a^Tobacco, alcohol, or drug (cocaine, opioids, stimulants, hallucinogens, sedatives, hypnotics, volatile solvents) use disorders.

Diabetic ketoacidosis [0.9 per 10,000 person-years (95% CI 0.7–1.12)], kidney complications [0.8 per 10,000 person-years (95% CI 0.7–1.1)], and neurological complications [0.8 per 10,000 person-years (95% CI 0.7–1.1)] were the specific diabetic complications with the highest incidence rates during the 10-year period (Table 2).

**Table 2:** Incidence rates of hospitalization for type 2 diabetic complications among women with gestational diabetes mellitus, within 10 years of delivery, Quebec, 1989 to 2018. N = 90,143.

	Total number of women	Incidence per 10,000 person-years (95% confidence interval)
Diabetes with any complication	283	3.8 (3.4–4.3)
Coma	21	0.3 (0.2–0.4)
Acidosis	69	0.9 (0.7–1.2)
Kidney complications	63	0.8 (0.7–1.1)
Ophthalmic	60	0.8 (0.6–1.0)
Neurological	61	0.8 (0.6–1.1)
Circulatory	52	0. 7 (0.5–0.9)
Other outcomes^a^	200	2.7 (2.3–3.1)

^a^Foot ulcer, multiple complications, and unspecified complications.

### Model performance for prediction of type 2 diabetic complications

The final models consisted of seven variables measured at the first GDM-affected pregnancy: maternal age, socioeconomic deprivation, substance use disorder, gestational age at delivery, SMM, previous pregnancy complications, and type of hypertensive disorder of pregnancy (Table 3; prediction model equation is provided in Table S4).

**Table 3:** Final prediction model with coefficients for prediction of type 2 diabetic complications among women with gestational diabetes mellitus, at 10 years of follow-up,

Variables	Model coefficients
**Maternal age**^**a**^	
Knot 1	Reference
Knot 2	− 0.0964
Knot 3	0.0805
**Socioeconomically deprived**	
No	Reference
Yes	0.7941
**Substance use**	
No	Reference
Yes	0.4916
**Gestational age at delivery**^**a**^	
Knot 1	Reference
Knot 2	0.0521
Knot 3	− 0.3401
**Severe maternal morbidity**	
No	Reference
Yes	2.7722
**Previous complications**	
No, multiparous	Reference
Yes, multiparous	0.2469
Primiparous	0.0447
**Hypertensive disorders of pregnancy**	
No HDP	Reference
Pre-existing or unspecified hypertension	− 1.610
Gestational hypertension	− 0.010
Pre-eclampsia/HELLP syndrome	1.690
Superimposed pre-eclampsia	− 9.7517
**Maternal age**^**a,b**^** severe maternal morbidity**	
Knot 1^b^ severe maternal morbidity (no)	Reference
Knot 2^b^ severe maternal morbidity (yes)	0.1121
Knot 3^b^ severe maternal morbidity (yes)	0.1359
**Maternal age**^**a,b**^** hypertensive disorders of pregnancy**	
Knot 1^b^ no hypertension	Reference
Knot 2^b^ pre-existing or unspecified hypertension	0.0820
Knot 3^b^ pre-existing or unspecified hypertension	− 0.1052
Knot 2^b^ gestational hypertension	0.0329
Knot 3^b^ gestational hypertension	− 0.0790
Knot 2^b^ pre-eclampsia/HELLP syndrome	− 0.0422
Knot 3^b^ pre-eclampsia/HELLP syndrome	0.0639
Knot 2^b^ superimposed pre-eclampsia	0.3724
Knot 3^b^ superimposed pre-eclampsia	− 0.2730

^a^Splines are fit for numerical variables and so coefficients are not provided in table.

^b^Severe maternal morbidity included conditions defined by the Canadian Perinatal Surveillance System^23^.

**Figure 2:**
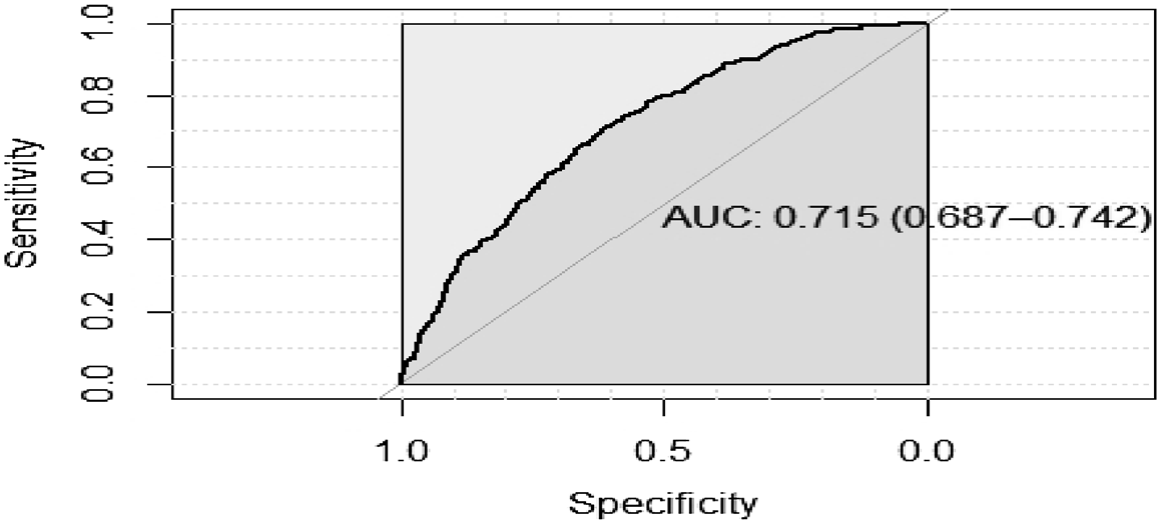
Area under the receiver operating characteristic curve [AUROC (95% confidence interval)] showing model discriminatory performance for 10-year prediction of type 2 diabetic complications.

**Figure 3:**
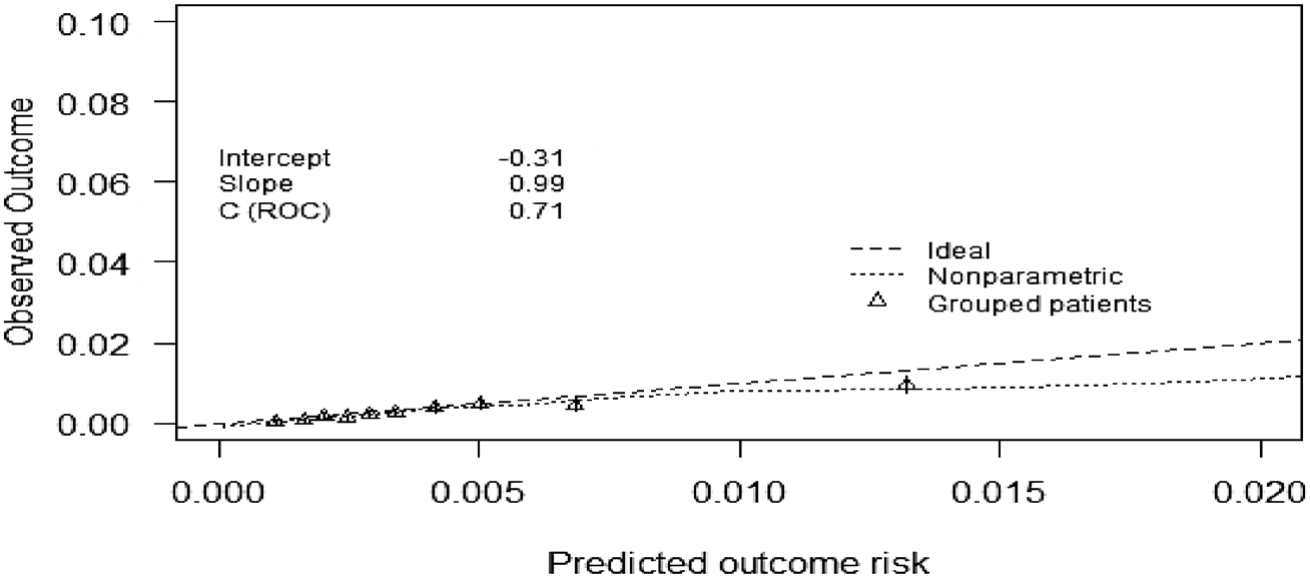
Calibration plots of observed versus predicted 10-year risks using deciles of predicted probability for type 2 diabetic complications.

The model had good discriminatory performance for the prediction of type 2 diabetic complications (AUROC 0.72, 95% CI 0.69–0.74) with a mean optimism of 0.0077 upon internal validation, indicating minimal overfitting (Fig. 2). Calibration performance was good with a slope of 0.99 and an intercept of − 0.31 (Fig. 3).

In the risk stratification table, the proportion of women with the primary outcome increased in each risk category, as the predicted risk of the outcome increased (Table 4). At the highest risk group (women with calculated risk of ≥ 0.03), 2.5% had the primary outcome whereas only 0.2% in the lowest risk group (women with calculated risk of < 0.006) had the outcome. The resulting LR+ in the highest risk group was 8.68 (95% CI 4.14–18.23) suggesting a moderate rule-in of type 2 diabetic complications in the top 0.3% of the women with the highest calculated predicted risk. However, the confidence interval was wide, due to low number of outcomes in this group (n = 7).

**Table 4:** Risk classification and stratification table for prediction of type 2 diabetic complications among women with gestational diabetes mellitus, at 10 years of follow-up.

Calculated risk probabilities groups^a^	N women in risk group (%)	N women with outcomes (%)	N women without outcomes (%)	Likelihood ratios
< 0.006	72,918 (80.9)	160 (0.2)	72,758 (99.8)	0.70 (0.63–0.77)
≥ 0.006–< 0.008	7595 (8.4)	33 (0.4)	7562 (99.6)	1.82 (1.33–2.48)
≥ 0.008–< 0.03	9367 (10.4)	83 (0.9)	9284 (99.1)	2.90 (2.42–3.48)
≥ 0.03	263 (0.3)	7 (2.5)	256 (97.5)	8.68 (4.14–18.23)
Total	90,143	283	89,860	

^a^Calculated predicted risks of developing the outcome grouped into different risk categories for stratification.

### Secondary analyses (prediction of outcome up to 29 years after delivery)

The incidence of the type 2 diabetic complications up to 29 years of follow-up was 9.0 (8.5–9.6) per 10,000 person-years, respectively (Table S3). Discriminatory performance decreased slightly, with AUROC of 0.63 (0.61–0.64).

## Discussion

In this study comprising 90,143 women with GDM, we developed Cox-proportional hazard models to predict the risk of developing type 2 diabetic complications occurring within 10 years of obstetric delivery. The incidence of hospitalization for diabetic complications in this timeframe was low, reflecting the young age of the cohort, and possibly good clinical care. However, our developed models consisting of readily measured variables at the time of birth showed moderate discriminative and calibration abilities for the prediction of type 2 diabetic complications after GDM pregnancy. Although external validation of the model is required, the AUROC of > 0.7 and LR+ of 8.68 in the highest risk category of ≥ 0.03 suggests that the model may eventually be used to reliably identify women at high risk of developing complicated diabetes.

Previous work has established a strong etiologic link between GDM and a future diagnosis of type 2 diabetes mellitus^4,5^. In a systematic review of 28 studies evaluating the risk of type 2 diabetes risk after GDM, the cumulative incidence of type 2 diabetes varied depending on time since delivery and ethnicity, and ranged from 2.6% to over 70% in included studies^2^. Our outcome of interest was hospitalization with type 2 diabetic complications within 10 years of delivery, reflecting more serious outcomes related to diabetes. The systematic review by Kim et al. also revealed that the cumulative incidence of type 2 diabetes increased over the first 5 years after delivery and plateaued after 10 years, supporting the chosen timeframe for our model^2^.

Previous models have focused on predicting type 2 diabetes after GDM, irrespective of the presence of diabetic complications^12,13^. A genetic risk score developed by Kwak et al. using data from 395 women with GDM reported a *C* statistic of 0.775 for predicting type 2 diabetes^12^. Another clinical model which included three types of lipid was developed by Lappas et al. in a cohort of 104 women and had *C* statistic ranging from 0.756–0.865^13^. These models did not include diabetic complications, which arguably have a more significant impact on women’s health, and would justify enhanced surveillance and early aggressive risk reduction therapy. Additionally, the small sample sizes used in these prior studies and the inclusion of variables not easily measured (e.g. genetic factors) lower the generalizability to routine clinical settings^18^.

Despite advances in our understanding of the association between pregnancy complications and long-term conditions, incorporation of pregnancy factors into existing risk scores has not improved net reclassification^27–29^. Possible reasons for the lack of success of previous models include their development in older populations and short length of follow-up, making them less suitable for women of reproductive age^30^. Developing prediction models among specific subpopulations, including postpartum women who have had a pregnancy complication, may circumvent some of these issues^12–14^. Despite the young age of individuals in our cohort, the predicted probability of type 2 diabetic complications at 10 years is substantial, indicating a robust model.

Late diagnosis of type 2 diabetes is often accompanied by development of diabetic complications, which is associated with healthcare expenditures three times greater than treatment of uncomplicated diabetes^6^. Clinical practice guidelines in Canada recommend performing a 75-g oral glucose tolerance test between 6 weeks and 6 months of delivery after GDM to identify type 2 diabetes early, with the goal of minimizing downstream sequelae^11^. Unfortunately, uptake of screening for type 2 diabetes after GDM remains low (< 50%), due to limited appreciation of risk, logistics of appointments and tests, and concerns of new mothers^11^. A shift in focus on screening strategies based on risk of more severe diabetes may allow for a greater mobilization of resources to a smaller segment of the population with GDM, particularly in lower resource settings. Our study shows that future type 2 diabetic complications can be predicted with moderate accuracy in women with GDM, using readily available clinical variables present in administrative datasets. The advantages of administrative data are that they are population-based records, and are larger than data from clinical settings, therefore our model can forecast future complications for health planning purposes^31^. The model requires external validation before ultimately being converted to an online risk calculator^18,32,33^ 8–10 to aid in counselling and triaging of resources in postpartum women with GDM. If the postpartum patient has a calculated high risk, the approach and follow-up would differ (e.g., endocrinology referral, early OGTT, intensive lifestyle approach) from that of a lower-risk patient (e.g., glycosylated hemoglobin, community follow-up, usual postpartum lifestyle recommendations). This individualized approach will help with the study, planning, and implementation of targeted preventive strategies at the population level.

Our study has several strengths. First, we used a large population-based cohort of women from Quebec, which represents more than one-quarter of the Canadian population with a diverse multi-ethnic population. We had sufficient sample size for model development and minimal overfitting as shown with internal validation. Second, our data allowed us to test short-term as well as long-term risks of the outcomes. We chose 10 years for the primary prediction period as this represents the time when up to 20% of individuals develop type 2 diabetes^2^. Furthermore, we followed model development methods recommended by experts, including the use of LASSO for final variable selection, rather than forward and backward selections which are more prone to bias^18^. Finally, we ensured transparency in modelling and reporting by following the TRIPOD statement^20^.

As is the case with many large administrative datasets, a downfall is the reliance on ICD codes to define predictors. In particular, the dataset lacked information on clinical parameters such as blood pressure, body mass index, lipid levels or blood glucose measurements which are commonly used in type 2 diabetes clinical prediction models. These continuous variables could potentially improve model discrimination as they increase heterogeneity between women^20^. Other markers of severity of GDM such as insulin levels are predictive of type 2 diabetes but were lacking in our dataset^2^. In addition, we lacked information on ethnicity and family history, which are known determinants of diabetes and its complications^2^. Our prediction models, therefore, need to be improved and externally validated before they can be considered for use in a clinical setting. Nonetheless, our study identified easily measured variables that contribute to the prediction of long-term type 2 diabetic complications after GDM. Our models should therefore be used as a foundation to improve upon risk classification after pregnancy.

Finally, it is important to note that the diagnosis of GDM has evolved over time^34^. However, in un-shown analyses, restricting our population to deliveries prior to 2010, corresponding with the International Association of Diabetes and Pregnancy Study Groups (IADPSG) diagnostic recommendations publication^34^, did not change model performance.

## Conclusion

Type 2 diabetic complications can be moderately predicted by our clinical model developed from administrative health data among individuals with a history of GDM. Future work is needed to improve the model performance and test the validity of the model in other datasets. After validation in external cohorts, women with GDM who are at high risk of developing type 2 diabetic complications can be earlier identified immediately after delivery. This will enable individualized planning and targeted approaches, such as endocrinology referral, early OGTT, and appropriate lifestyle modifications, to prevent future type 2 diabetic complications. Simple models to predict diabetic complications such as ours have the potential to help to develop a rational risk-based approach to screening and surveillance after GDM.

## Supplementary Information

Supplementary Tables.

## Data Availability

The data that support the findings of this study are available from Institut de la statistique du Québec but restrictions apply to the availability of these data, which were used under license for the current study, and so are not publicly available. Data are however available from Institut de la statistique du Québec upon reasonable request and with permission.
