# Supplementary material for: Development and internal validation of a model to predict type 2 diabetic complications after gestational diabetes
Source: Sci Rep. 2022 Jun 20;12:10377. doi: 10.1038/s41598-022-14215-9 (PMC9209541; doi:10.1038/s41598-022-14215-9)
Supplement: Supplementary file 1 — Supplementary Tables. [file 41598_2022_14215_MOESM1_ESM.docx]

**Table S1.** Diagnostic codes for primary outcome of gestational diabetes and type 2 diabetic complications

|  | International Classification of Diseases 9^th^ revision,  before 2006 | International Classification of Diseases 10^th^ revision,  beginning in 2006 |
| --- | --- | --- |
| **Gestational diabetes** | 648.8 | O24 |
| **Type 2 diabetes**  **With coma** | 250.2 - 250.3 | E11.0 |
| **With acidosis:** | 250.1 | E11.1 (Includes E11.10- E11.12) |
| ketoacidosis |  | E11.10 |
| lactic acidosis |  | E11.11 |
| ketoacidosis with lactic acidosis |  | E11.12 |
| **With kidney complications:** | 250.4 | E11.2 (Includes E11.20- E11.28) |
| incipient diabetic nephropathy |  | E11.20 |
| established or advanced kidney disease |  | E11.23 |
| other specified kidney complication not elsewhere classified |  | E11.28 |
| **With ophthalmic complications:** | 250.5 | E11.3 (Includes E11.30- E11.38) |
| background retinopathy |  | E11.30 |
| preproliferative retinopathy |  | E11.31 |
| proliferative retinopathy |  | E11.32 |
| other retinopathy |  | E11.33 |
| advanced ophthalmic disease |  | E11.36 |
| other specified ophthalmic complication not elsewhere classified |  | E11.38 |
| **With neurological complications:** | 250.6 | E11.4 (Includes E11.40- E11.42) |
| mononeuropathy |  | E11.40 |
| polyneuropathy |  | E11.41 |
| autonomic neuropathy |  | E11.42 |
| **With circulatory complications:** | 250.7 | E11.5 (Includes E11.50- E11.52) |
| peripheral angiopathy |  | E11.50 |
| peripheral angiopathy with gangrene |  | E11.51 |
| certain circulatory complications |  | E11.52 |
| **With other specified complications:** | 250.8 | E11.6 |
| musculoskeletal and connective tissue complication |  | E11.60 |
| skin and subcutaneous tissue complication |  | E11.61 |
| periodontal complication |  | E11.62 |
| hypoglycaemia |  | E11.63 |
| poor control |  | E11.64 |
| other specified complication, not elsewhere classified |  | E11.68 |
| **With multiple complications:** |  | E11.7 |
| foot ulcer (angiopathic)(neuropathic) |  | E11.70 |
| foot ulcer (angiopathic) (neuropathic) with gangrene |  | E11.71 |
| multiple other complications |  | E11.78 |
| **With unspecified complication** | 250.9 |  |

Type 2 diabetic complications was defined as diagnosis with type 2 diabetes and one or more of the conditions listed in the table.

**Table S2.** TRIPOD Checklist: Prediction Model Development and Validation

| **Section/Topic** |  |  | **Checklist Item** | **Page** |
| --- | --- | --- | --- | --- |
| **Title and abstract** | | | | |
| Title | 1 | D;V | Identify the study as developing and/or validating a multivariable prediction model, the target population, and the outcome to be predicted. | 1 |
| Abstract | 2 | D;V | Provide a summary of objectives, study design, setting, participants, sample size, predictors, outcome, statistical analysis, results, and conclusions. | 3 |
| **Introduction** | | | | |
| Background and objectives | 3a | D;V | Explain the medical context (including whether diagnostic or prognostic) and rationale for developing or validating the multivariable prediction model, including references to existing models. | 5 |
|  | 3b | D;V | Specify the objectives, including whether the study describes the development or validation of the model or both. | 5 |
| **Methods** | | | | |
| Source of data | 4a | D;V | Describe the study design or source of data (e.g., randomized trial, cohort, or registry data), separately for the development and validation data sets, if applicable. | 6 |
|  | 4b | D;V | Specify the key study dates, including start of accrual; end of accrual; and, if applicable, end of follow-up. | 6 |
| Participants | 5a | D;V | Specify key elements of the study setting (e.g., primary care, secondary care, general population) including number and location of centres. | 6 |
|  | 5b | D;V | Describe eligibility criteria for participants. | 7 |
|  | 5c | D;V | Give details of treatments received, if relevant. | n/a |
| Outcome | 6a | D;V | Clearly define the outcome that is predicted by the prediction model, including how and when assessed. | 7& Table S1 |
|  | 6b | D;V | Report any actions to blind assessment of the outcome to be predicted. | n/a |
| Predictors | 7a | D;V | Clearly define all predictors used in developing the multivariable prediction model, including how and when they were measured. | 7-8 |
|  | 7b | D;V | Report any actions to blind assessment of predictors for the outcome and other predictors. | n/a |
| Sample size | 8 | D;V | Explain how the study size was arrived at. | 10 |
| Missing data | 9 | D;V | Describe how missing data were handled (e.g., complete-case analysis, single imputation, multiple imputation) with details of any imputation method. | n/a |
| Statistical analysis methods | 10a | D | Describe how predictors were handled in the analyses. | 7-8 |
|  | 10b | D | Specify type of model, all model-building procedures (including any predictor selection), and method for internal validation. | 7-9 |
|  | 10c | V | For validation, describe how the predictions were calculated. | n/a |
|  | 10d | D;V | Specify all measures used to assess model performance and, if relevant, to compare multiple models. | 9-10 |
|  | 10e | V | Describe any model updating (e.g., recalibration) arising from the validation, if done. | n/a |
| Risk groups | 11 | D;V | Provide details on how risk groups were created, if done. | 9 |
| Development vs. validation | 12 | V | For validation, identify any differences from the development data in setting, eligibility criteria, outcome, and predictors. | n/a |
| **Results** | | | | |
| Participants | 13a | D;V | Describe the flow of participants through the study, including the number of participants with and without the outcome and, if applicable, a summary of the follow-up time. A diagram may be helpful. | 10-11/Figure 1 |
|  | 13b | D;V | Describe the characteristics of the participants (basic demographics, clinical features, available predictors), including the number of participants with missing data for predictors and outcome. | 10-11/Table 1 |
|  | 13c | V | For validation, show a comparison with the development data of the distribution of important variables (demographics, predictors and outcome). | n/a |
| Model development | 14a | D | Specify the number of participants and outcome events in each analysis. | 10-11 |
|  | 14b | D | If done, report the unadjusted association between each candidate predictor and outcome. | n/a |
| Model specification | 15a | D | Present the full prediction model to allow predictions for individuals (i.e., all regression coefficients, and model intercept or baseline survival at a given time point). | Table 3 |
|  | 15b | D | Explain how to use the prediction model. | n/a |
| Model performance | 16 | D;V | Report performance measures (with CIs) for the prediction model. | 11-12/Table 4-4/Figures 2&3 |
| Model-updating | 17 | V | If done, report the results from any model updating (i.e., model specification, model performance). | n/a |
| **Discussion** | | | | |
| Limitations | 18 | D;V | Discuss any limitations of the study (such as nonrepresentative sample, few events per predictor, missing data). | 15 |
| Interpretation | 19a | V | For validation, discuss the results with reference to performance in the development data, and any other validation data. | n/a |
|  | 19b | D;V | Give an overall interpretation of the results, considering objectives, limitations, results from similar studies, and other relevant evidence. | 12-16 |
| Implications | 20 | D;V | Discuss the potential clinical use of the model and implications for future research. | 12-16 |
| **Other information** | | | | |
| Supplementary information | 21 | D;V | Provide information about the availability of supplementary resources, such as study protocol, Web calculator, and data sets. | n/a |
| Funding | 22 | D;V | Give the source of funding and the role of the funders for the present study. | 17 |

**Table S3.** Incidence rates of hospitalization for type 2 diabetic complications for women with gestational diabetes mellitus, within 29 years of delivery, Quebec, 1989 to 2018. N = 90,143

|  |  | | |
| --- | --- | --- | --- |
|  | | Number of women | Incidence per 10,000 person-years (95% confidence interval) |
| Diabetes with any complications | | 1025 | 9.0 (8.5-9.6) |
| Coma | | 33 | 0.3 (0.2-0.4) |
| Acidosis | | 114 | 1.0 (0.8-1.21) |
| Kidney complications | | 160 | 1.4 (1.2-1. 7) |
| Ophthalmic | | 132 | 1.2 (1.0-1.4) |
| Neurological | | 148 | 1.3 (1.1-1.6) |
| Circulatory | | 99 | 0.9 (0.7-1.1) |
| Other outcomes* | | 770 | 6.9 (6.4-7.4) |

^†^Other outcomes include foot ulcer, multiple complications, and unspecified complications

**Table S4.** Prediction model formula

|  |  |
| --- | --- |
| Log hazard ratio =   β_0_ + (β_1_ × maternal age) + (β_2_ × socioeconomically deprived) + (β_3_ × substance use) + (β_4_ × gestational age at delivery) + (β_5_ × severe maternal morbidity) + (β_6_ × previous pregnancy complications) + (β_7_ × hypertensive disorder of pregnancy group) + (β_8_ × maternal age × severe maternal morbidity) + (β_9_ × maternal age ×  hypertensive disorder of pregnancy group) | |

^†^β represent the coefficients provided in Table 3.
